# DNA vaccination induced protective immunity against SARS CoV-2 infection in hamsterss

**DOI:** 10.1371/journal.pntd.0009374

**Published:** 2021-05-27

**Authors:** Kit Man Chai, Tsai-Teng Tzeng, Kuan-Yin Shen, Hung-Chun Liao, Jhe-Jhih Lin, Mei-Yu Chen, Guann-Yi Yu, Horng-Yunn Dou, Ching-Len Liao, Hsin-Wei Chen, Shih-Jen Liu

**Affiliations:** 1 National Institute of Infectious Diseases and Vaccinology, National Health Research Institutes, Miaoli, Taiwan; 2 Department of Life Sciences, National Tsing Hua University, Hsinchu, Taiwan; 3 Graduate Institute of Biomedical Sciences, China Medical University, Taichung, Taiwan; 4 Graduate Institute of Medicine, College of Medicine, Kaohsiung Medical University, Kaohsiung, Taiwan; The University of Hong Kong, HONG KONG

## Abstract

The development of efficient vaccines against COVID-19 is an emergent need for global public health. The spike protein of severe acute respiratory syndrome coronavirus 2 (SARS-CoV-2) is a major target for the COVID-19 vaccine. To quickly respond to the outbreak of the SARS-CoV-2 pandemic, a nucleic acid-based vaccine is a novel option, beyond the traditional inactivated virus vaccine or recombinant protein vaccine. Here, we report a DNA vaccine containing the spike gene for delivery via electroporation. The spike genes of SARS-CoV and SARS-CoV-2 were codon optimized for mammalian cell expression and then cloned into mammalian cell expression vectors, called pSARS-S and pSARS2-S, respectively. Spike protein expression was confirmed by immunoblotting after transient expression in HEK293T cells. After immunization, sera were collected for antigen-specific antibody and neutralizing antibody titer analyses. We found that both pSARS-S and pSARS2-S immunization induced similar levels of antibodies against S2 of SARS-CoV-2. In contrast, only pSARS2-S immunization induced antibodies against the receptor-binding domain of SARS-CoV-2. We further found that pSARS2-S immunization, but not pSARS-S immunization, could induce very high titers of neutralizing antibodies against SARS-CoV-2. We further analyzed SARS-CoV-2 S protein-specific T cell responses and found that the immune responses were biased toward Th1. Importantly, pSARS2-S immunization in hamsters could induce protective immunity against SARS-CoV-2 challenge *in vivo*. These data suggest that DNA vaccination could be a promising approach for protecting against COVID-19.

## Author summary

SARS-CoV-2 continues to threaten global health, and development of SARS-CoV-2 vaccine is an urgent priority to halt COVID-19 epidemic. Here, we developed DNA vaccines containing the spike gene from SARS-CoV-2 and delivered via electroporation. We observed that the DNA vaccine induced a long-lasting humoral response against SARS-CoV-2, and the elicited serum antibodies efficiently blocked the binding of SARS-CoV-2 RBD to its entry receptor (ACE2). Importantly, the DNA vaccine induced strong Th1-biased immune responses against SARS-CoV-2, which could provide protective effect with low side effects. Finally, we demonstrated that SARS-CoV-2 DNA vaccine conferred protective efficacy against SARS-CoV-2 infection in Syrian hamsters. Our findings suggested that DNA vaccination could be a useful approach for controlling COVID-19 pandemic in near future.

## Introduction

The emerging infectious disease COVID-19, caused by severe acute respiratory syndrome-associated coronavirus 2 (SARS-CoV-2), has had significant economic impacts in countries affected by the disease outbreak in 2019–2020 [1]. Globally, there have been more than 118 million confirmed cases of COVID-19, and more than 2.6 million people died of COVID-19, by March 2021 [2]. The global case fatality rate is approximately 2.2%. Although the mortality rate of SARS-CoV-2 is lower than that of MERS-CoV and SARS-CoV infection, its transmissibility is higher. Several COVID-19 vaccines have been approved for emergent used in December 2020, but the COVID-19 pandemic remains an international threat as of this writing.

Spike (S) protein is the coronavirus surface protein that is responsible for the processes of virus attachment to the host receptor angiotensin-converting enzyme 2 (ACE2), cell entry, and virus-cell membrane fusion to release viral RNA into host cells. Among the structural proteins of SARS-CoV, the spike protein is the dominant antigen that induces neutralizing antibodies [3]. Based on previous studies on SARS and MERS, S protein-based vaccines have been proven to induce neutralizing antibodies and T cell immune responses to coronaviruses and protect animals from virus challenge [4, 5]. Due to the high immunogenic of S protein, it could be a potential target for SARS-CoV-2 vaccine development [5–7]. COVID-19 vaccine candidates could be developed using inactivated virus, recombinant or synthetic viral components, recombinant virus, or viral mRNA or DNA. The latter approach is particularly attractive because viral DNA can be produced quickly and easily delivered worldwide without a cold chain system. Moreover, fully synthetic DNA derived from the sequence encoding the viral protein, could induce both humoral and cell-mediated immune responses against pathogens [8, 9].

The DNA vaccine is an ideal vaccine platform with several advantages, including easy design and production, stability at a range of temperatures, and low production cost. Hence, the DNA vaccine platform is suitable for rapid and large-scale manufacturing during infectious disease outbreaks. Previous studies have reported that DNA vaccines can effectively stimulate cellular and humoral responses against pathogens in challenge models [10]. Furthermore, recent clinical studies indicated that DNA vaccines are safe and effective candidates for treating or preventing infectious diseases, such as HIV-1, Zika virus, Ebola virus, MERS-CoV, and influenza viruses [11]. As the COVID-19 pandemic has spread globally and severely, recent studies reported that DNA vaccines elicited antigen-specific T cell responses and neutralizing antibodies and further protected animals against SARS-CoV-2 challenge [12, 13].

The major challenge of DNA vaccines is the poor efficiency of DNA delivery into cells for antigen expression and consequently poor efficacy of the vaccines. To increase the DNA delivery efficiency, physical methods or chemical methods can be used. The physical methods include high-pressure air stream (i.e., Biojector), gold particle-coated DNA delivery by gene gun, microneedle array and electroporation (EP). The chemical methods include liposomes, virosomes, nanoparticles and cell-penetrating peptides [14]. In the Zika virus outbreak in 2015, a Zika DNA vaccine delivered via electroporation was developed into a phase 1 clinical trial within 7 months [15]. EP combined with DNA vaccination greatly increases the efficacy of DNA vaccines [16–18]. Because of the successful results of animal experiments after DNA vaccination with EP, many different electroporation devices for humans have been developed, including Cellectra® (Inovio Inc., USA) and TriGrid® (Ichor Medical Systems, USA).

This study describes a DNA vaccination with EP that can induce neutralizing antibody and Th1-biased immune responses. Hamsters immunized with this technique generated neutralizing antibodies against SARS-CoV-2. Furthermore, the immunized hamsters exhibited protective immunity in a SARS-CoV-2 virus challenge.

## Methods

### Ethics statement

All animal experimental protocols were approved by the Institutional Animal Care and Use Committee (IACUC) of the NHRI (Protocol No: NHRI-IACUC-109077-A).

### Cell lines

Human embryonic kidney cell line HEK293T was cultured in Dulbecco’s modified Eagle’s medium (DMEM, GIBCO) supplemented with 10% heat-inactivated fetal bovine serum (FBS, HyClone), 100 U/mL penicillin/streptomycin (GIBCO) and 2 mM L-glutamine (GIBCO). Vero cells were cultured in M199 medium (GIBCO) with 5% FBS at 37°C.

### Virus titration

SARS-CoV-2 variants (hCoV-19/Taiwan/4/2020 and hCoV-19/Taiwan/78/2020 (D614G variant)) were obtained from the Centers for Disease Control (CDC) in Taiwan. The virus was amplified in Vero cells grown in M199 medium supplemented with 2 μg/mL TPCK-trypsin (Sigma) at 37°C. The virus titer was determined in terms of the 50% tissue culture infectious dose (TCID_50_) using a standard method [19]. Briefly, Vero cells were seeded (2.4×10^4^ cells/per well) in 96-well plates and cultured in M199 medium with 5% FBS at 37°C for 24 h to form a monolayer. The next day, serial 10-fold dilutions were prepared, and the diluted virus (100 μL/well) was added onto Vero cell monolayers with eight replicates per dilution. After 4 days of incubation at 37°C, the virus-induced cytopathic effects (CPE) in each well were recorded, and the results are expressed as TCID_50_/mL according to the method of Reed and Muench. All experiments with SARS-CoV-2 were conducted in the biosafety level 3 (BSL-3) laboratory and were approved by the Taiwan CDC.

### Plasmid construction and characterization

The DNA sequences encoding full-length SARS-Spike (GenBank accession number DQ412574) and SARS-CoV-2 spike genes (GenBank accession number: MN908947) were optimized for mouse codon usage and synthesized by GenScript Biotech. Different fragments of S (tRBD, tRBDTM, tSARS2-S, tSdTM) were also constructed and amplified individually by PCR. All genes were subcloned into the clinically used vector pVAX1 with Kozak sequence incorporated into the 5’ end of the genes. The plasmid was transformed into DH5α *E*. *coli* for plasmid amplification. Plasmids were extracted and purified using an endotoxin-free Qiagen column system (EndoFree Plasmid Mega Kit).

### Transient expression and Western blot

HEK293T cells were transfected with the indicated DNA plasmids using PolyJet™ reagent (SignaGen Laboratories) following the manufacturer’s protocol. At 24 hours post transfection, cell lysates were harvested and subjected to electrophoresis on 8% SDS-PAGE. The proteins were then transferred to PVDF membranes and blotted with rabbit anti-Spike polyclonal antibody (40592-T62, Sino Biological). Horseradish peroxidase (HRP)-conjugated anti-rabbit antibodies were used as the secondary antibody. Specific proteins on the membrane were visualized by ECL reagent (Thermo Scientific).

### Animal immunization

BALB/c, C57BL/6 mice and Syrian hamsters were obtained from the National Laboratory Animal Breeding and Research Center (Taipei, Taiwan). Mice or hamsters were used between 6 and 12 weeks of age. Anesthetized mice or hamsters were vaccinated with 100 μL of solution containing indicated DNA in a 3-week interval, followed by electroporation with a BTX electroporator (ECM830) using two-needle array electrodes (5-mm diameter (BTX 45–0121)). Intramuscular electroporation was performed at 75 V constant voltage with 10 pulses at 50 msec/pulse and 100-msec intervals between pulses. Blood samples of mice and hamsters were collected by submandibular or retroorbital blood samplings, respectively. All animals were housed at the Laboratory Animal Center of the National Health Research Institutes (NHRI) and maintained in accordance with institutional animal care protocols.

### Immunoassay

The presence of S-specific antibodies in sera was determined by ELISA. Briefly, 50 μL of 4 μg/mL recombinant protein (Sino Biological) in 0.1 M carbonate buffer (pH 9.5) was coated onto 96-well microplates by overnight incubation at 4°C. Coated plates were washed twice with 0.05% Tween 20 in PBS and then blocked with 3% BSA in PBS at room temperature for 2 hours. Diluted sera from immunized animals were applied to wells at room temperature for 2 hours. Following the addition of HRP-conjugated goat anti-mouse IgG (Thermo Scientific) or HRP-conjugated goat anti-hamster IgG (Arigo Biolaboratorie), the assay was developed by using SureBlue TMB 1-Component Peroxidase Substrate (KPL). The absorbance was measured using an ELISA reader at 450 nm.

### Neutralization of SARS-CoV-2 virus infection

Vero cells were seeded (2.4×10^4^ cells/well) in 96-well plates for 24 h to form a monolayer. Preimmune sera and antisera against SARS-CoV-2 S protein were pretreated at 56°C for 30 minutes to destroy heat-labile nonspecific viral inhibitory substances. The sera were diluted to an initial dilution of 1/20 with M199 medium, added into a well containing 200 TCID_50_ of SARS-CoV-2 virus in a volume of 0.2 mL, and then incubated at 37°C for 2 h. Subsequently, the virus-serum mixture was inoculated onto Vero cell monolayers and incubated at 37°C. Quadruplicates were prepared for each serum dilution. The CPE characteristics in each well were recorded after 4–5 days of incubation. The neutralization titer was proportional to the highest dilution of serum that prevented infection of 50% of quadruplicate inoculations.

### ACE2 competition ELISA

ACE2 competition ELISA was performed by using the Anti-SARS-CoV-2 Neutralizing Antibody Titer Serologic Assay Kit (ACROBiosystems) according to the recommended protocol. Briefly, 96-well plates were coated with 0.5 μg/mL SARS-CoV-2-S RBD protein overnight at 4°C. The plate was washed and blocked with blocking buffer at 37°C for 1.5 hours. After three washes, biotinylated human ACE2 (0.12 μg/mL) was added to the wells, followed by dilution of the serum samples and incubation at 37°C for 1 hour. To generate a standard curve, anti-SARS-CoV-2 neutralizing antibody as provided by the kit was used as a reference. The plate was washed, and streptavidin-HRP working solution was added to each well for 1 hour at 37°C. The plate was then washed, and the assay was developed by incubation with TMB substrate working solution at 37°C for 20 min. The reaction was stopped with stop solution provided. The absorbance was measured using an ELISA reader at 450 nm. The competitive activity of serum antibodies was expressed as the corresponding level of reference antibody.

### Cytokine production assay

T cell responses were assessed using cytokine ELISA. Splenocytes from immunized mice were plated at a density of 5×10^6^ cells per well in 24-well plates. The cells were stimulated with 5 μg/mL recombinant SARS-CoV-2 Spike protein (ACROBiosystems) at 37°C for 3 days. The supernatant was harvested and assayed for cytokine production. Mouse IL-2, IL-5, IL-13 and IFN-γ were quantitated by ELISA using the matching antibody set (Invitrogen) in accordance with the manufacturer’s instructions.

### Animal challenge

Syrian hamsters (n = 8 per group) were intramuscularly immunized by needle injection with plasmid DNA (100 μg/animal), followed by BTX electroporation as mentioned above. At four weeks after the last vaccination, the hamsters were challenged intranasally with 10^5^ TCID_50_ SARS-CoV-2 (hCoV-19/Taiwan/4/2020) in 50 μL under isoflurane anesthesia. Their body weight (n = 4 per group) was recorded every day for 9 days after challenge. Four hamsters in each group were sacrificed at day 3 after challenge for viral load quantification. To determine the viral load in the lung, left lung tissues were homogenized in 2 mL of PBS using a gentleMACS® Dissociator (Miltenyi Biotec). After centrifugation at 600 x g for 5 minutes, the clarified supernatant was harvested for live virus titration (TCID_50_ assay) and viral RNA quantification.

### Quantification of viral RNA load

Clarified supernatant of homogenized left lung tissue from SARS-CoV-2-infected hamsters was harvested for viral load detection. RNA extraction was carried out on tissue supernatant lysed with TRIzol LS (Ambion), and 10 ng of the RNA was used as a template for RT-qPCR reactions. RT-qPCR was performed on a QuantStudio 6 Flex Real-Time PCR System (ABI) using the KAPA PROBE FAST Universal One-Step qRT-PCR kit (KR1282, Roche) with primers and probes specific for SARS-CoV-2 (E_Sarbeco Forward: ACAGGTACGTTAATAGTTAATAGCGT, E_Sarbeco Reverse: ATATTGCAGCAGTACGCACACA, E_Sarbeco Probe: FAM-ACACTAGCCATCCTTACTGCGCTTCG-BHQ1) [20].

### Statistical analysis

Statistical data were generated using GraphPad Prism software. The statistical significance of differential findings between experimental groups was determined by the two-tailed Mann-Whitney test. Differences were considered statistically significant if the p value was ≤ 0.05.

## Results

### Plasmid construction and antigen expression of vaccine candidates

**Fig 1:**
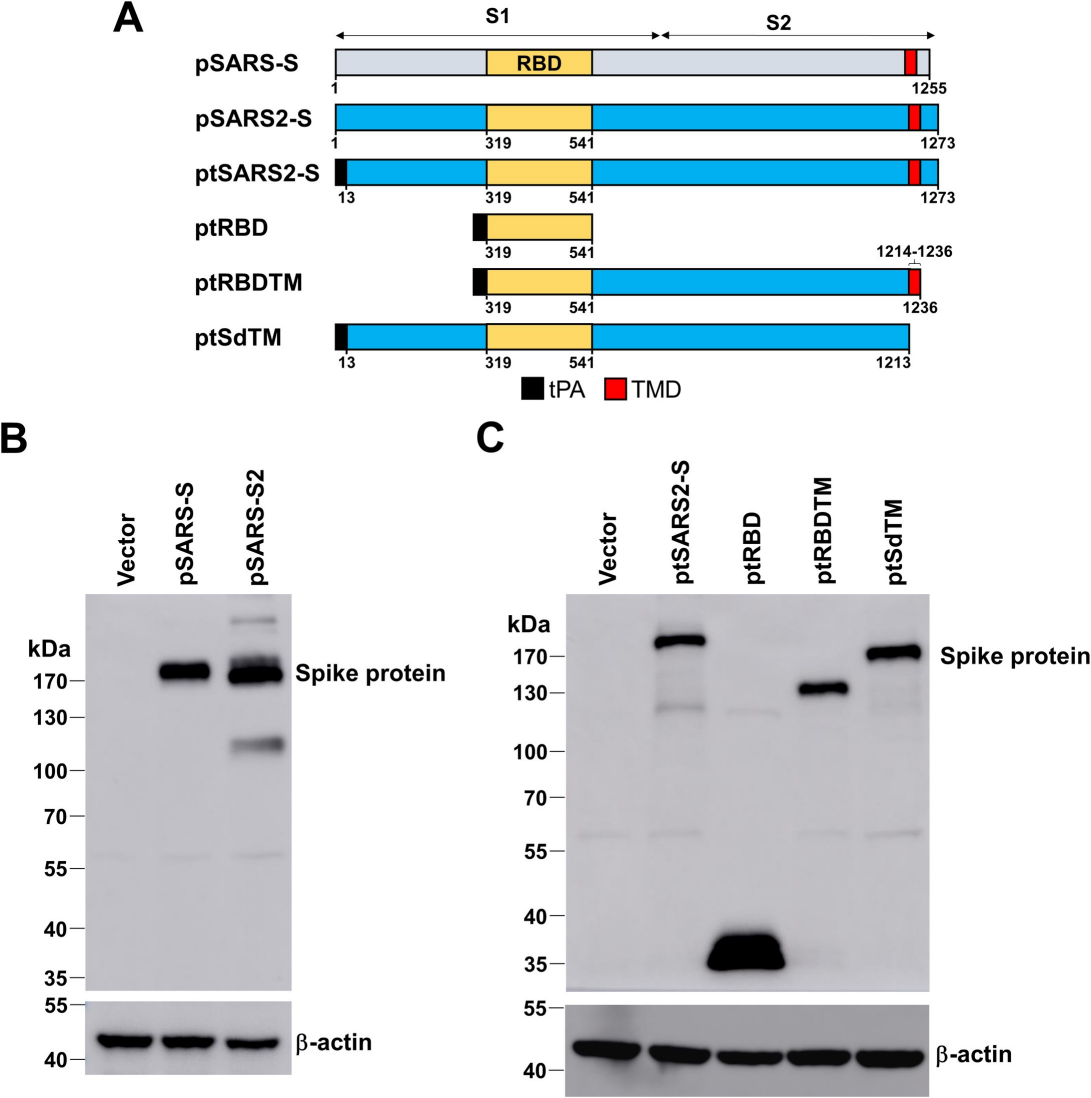
Design and expression of SARS-CoV and SARS-CoV-2 spike construct variants. (A) Schematic diagram of SARS-CoV and SARS-CoV-2 spike construct variants. tPA, leader sequence from tissue-plasminogen activator; TM, transmembrane domain. (B, C) Western blot analysis of spike protein. HEK293T cells were transfected with the indicated plasmids (vector, pSARS-S, pSARS2-S, and S variants fused with tPA leader sequence). The cell lysates were collected and probed with anti-Spike antibody, and anti-β-actin antibody was used as an internal control.

Five variants of SARS-CoV-2 and one SARS-CoV construct containing different fragments of spike protein-encoding DNA were generated (Fig 1A). Because the antigen expression strongly correlated to vaccine efficacy, we designed different spike protein fragments, including RBD (aa_319_-aa_541_), RBD to TM (aa_319_-aa_1236_) or Spike with transmembrane domain (TM) deletion (aa_13_-aa_1213_), with human tissue plasminogen activation (tPA) leader sequence that may increase the secretion of antigens [21]. The RBD to TM domain can keep S2 domain that is important to form six-helix structure for cell fusion [22, 23]. These constructs were as follows: full-length S of SARS-CoV (pSARS-S) and SARS-CoV-2 (pSARS2-S), full-length spike with the leader sequence of tissue-plasminogen activator (ptSARS2-S), RBD region (ptRBD), RBD to transmembrane domain (ptRBDTM) and spike with a deletion of the transmembrane domain (ptSdTM). Vector, pSARS-S and pSARS2-S were transfected into and expressed in HEK293T cells. The cell lysates were analyzed by SDS-PAGE. The full-length S could be detected at the corresponding molecular weight (Fig 1B). The variant S of SARS2-CoV-2 with a leader sequence of tissue-plasminogen activator was detected with an anti-Spike polyclonal antibody. The data showed that each variant was observed at the expected molecular weight (Fig 1C). The expression levels of ptSARS2-S, ptRBDTM and ptSdTM variants were similar, while ptRBD expression was higher in all variants.

### Immunogenicity of vaccine candidates

**Fig 2:**
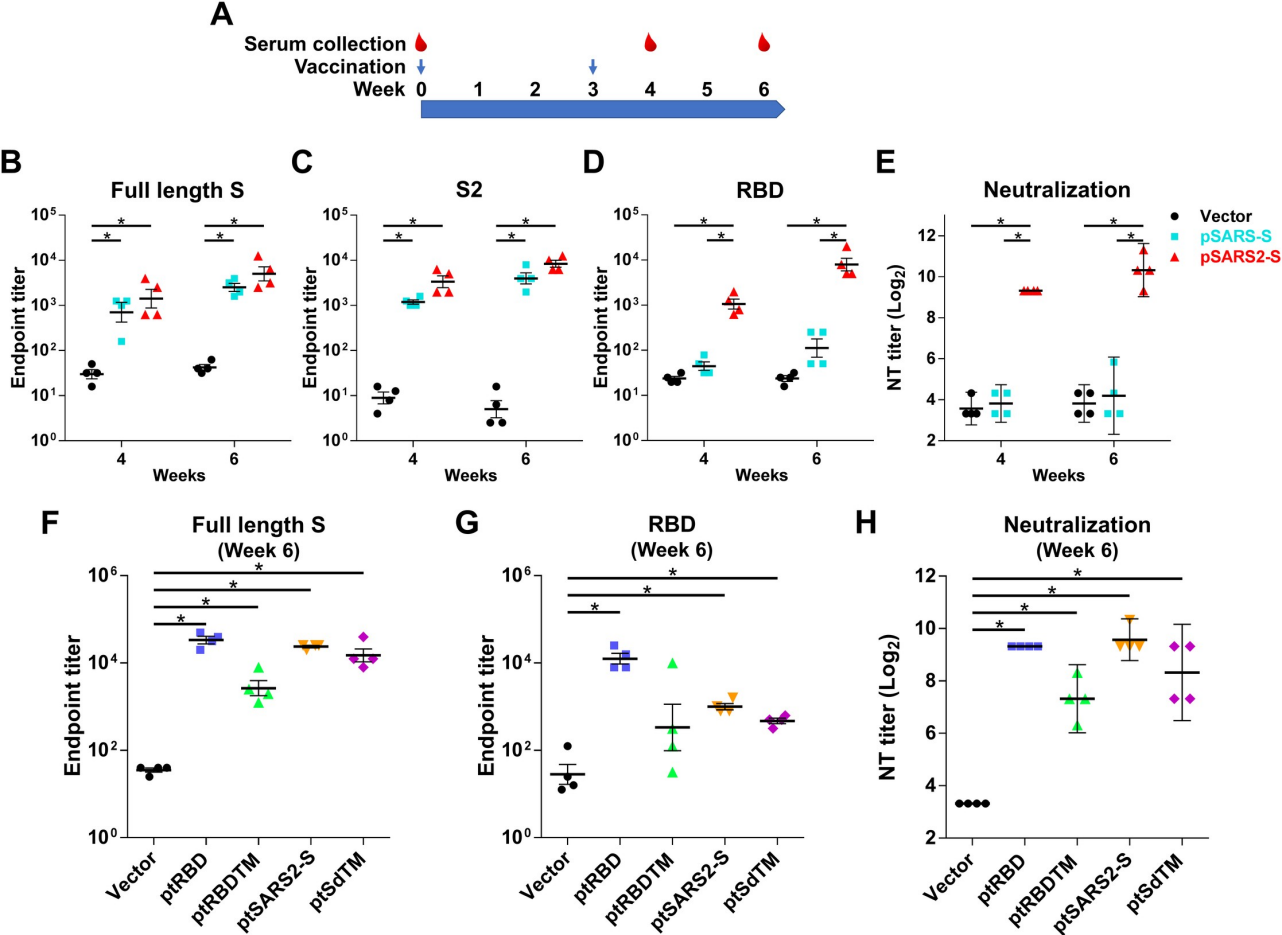
Antibody response in mice after immunization with SARS-CoV and SARS-CoV-2 S DNA vaccines. (A) BALB/c mice (n = 4 per group) were intramuscularly immunized twice at a 3-week interval with 100 μg of indicated plasmid, followed by electroporation. Serum samples were collected at the indicated time points after the first immunization. (B-D, F, G) Antibodies against the SARS-CoV-2 full-length spike protein, S2 region and RBD were evaluated by ELISA. (E, H) Vaccine-induced neutralizing antibody against SARS-CoV-2 was evaluated by neutralization assay. Antibody titers are presented as the mean ± SEM, and neutralization titers are expressed as the geometric mean with a 95% confidence interval. *p<0.05 by the Mann-Whitney test.

To examine the immunogenicity of different variants, BALB/c mice were intramuscularly injected twice at a 3-week interval with 100 μg of vector, pSARS-S and pSARS2-S followed by *in vivo* electroporation (Fig 2A). Sera were collected at week 4 and week 6 after the first immunization. The data showed that sera of pSARS-S and pSARS2-S immunized animals could recognize both the full-length S and S2 regions of SARS-CoV-2 with similar IgG antibody titers (Fig 2B and 2C). In contrast, sera from pSARS2-S immunized animals could raise high anti-RBD (SARS-CoV-2) antibody titers at week 4 and week 6 (Fig 2D), compared to pSARS-S group. Accordingly, sera of pSARS2-S immunized animals but not pSARS-S immunized animals could neutralize SARS-CoV-2 infection (Fig 2E). The geometric mean titers (log_2_) of neutralizing antibody in the pSARS2-S immunization group at weeks 4 and 6 were 9.3 and 10.3, respectively. These results indicated that pSARS-S immunization cannot induce cross-neutralization antibodies against SARS-CoV-2 infection. The RBD-specific antibodies were critical for neutralizing activity against SARS-CoV-2 infection. The full-length S of SARS-CoV-2 can indeed induce high levels of neutralizing antibody titers against SARS-CoV-2 infection.

To further investigate whether replacement of the leader sequence can increase protein secretion, we used a leader sequence from tissue-plasminogen activator to fuse different variants of SARS-CoV-2 S protein. All variants contain the RBD region of the SARS-CoV-2 S protein. After two doses of DNA immunization, the sera collected at week 4 and week 6 were analyzed for IgG antibody and neutralizing antibody titers. Immunization with ptSARS2-S induced higher antibody titers against full-length S protein at week 4 than ptRBD, ptRBDTM and ptSdTM immunization (S1A Fig). The anti-RBD antibody titers of ptRBD immunization were higher than those of ptSARS2-S immunization (1496.2 vs. 530.9, p = 0.057) at week 4 (S1B Fig). However, ptRBD and ptSARS2-S immunization induced similar levels of neutralizing antibodies at week 4 (S1C Fig). The same results were observed in the week 6 sera analysis; ptRBD immunization induced higher levels of anti-RBD antibodies compared to ptSARS2-S immunization (12589.3 vs. 1000.0, p = 0.028) but the same levels of neutralizing antibodies (9.3 vs. 9.6 (log_2_)) (Fig 2F, 2G and 2H). Because the tPA leader sequence-fused variants did not induce higher neutralizing antibody titers than pSARS2-S immunization, we used pSARS2-S for further investigation.

### Competitive binding of serum antibodies and ACE2 to SARS-CoV-2 RBD

**Fig 3:**
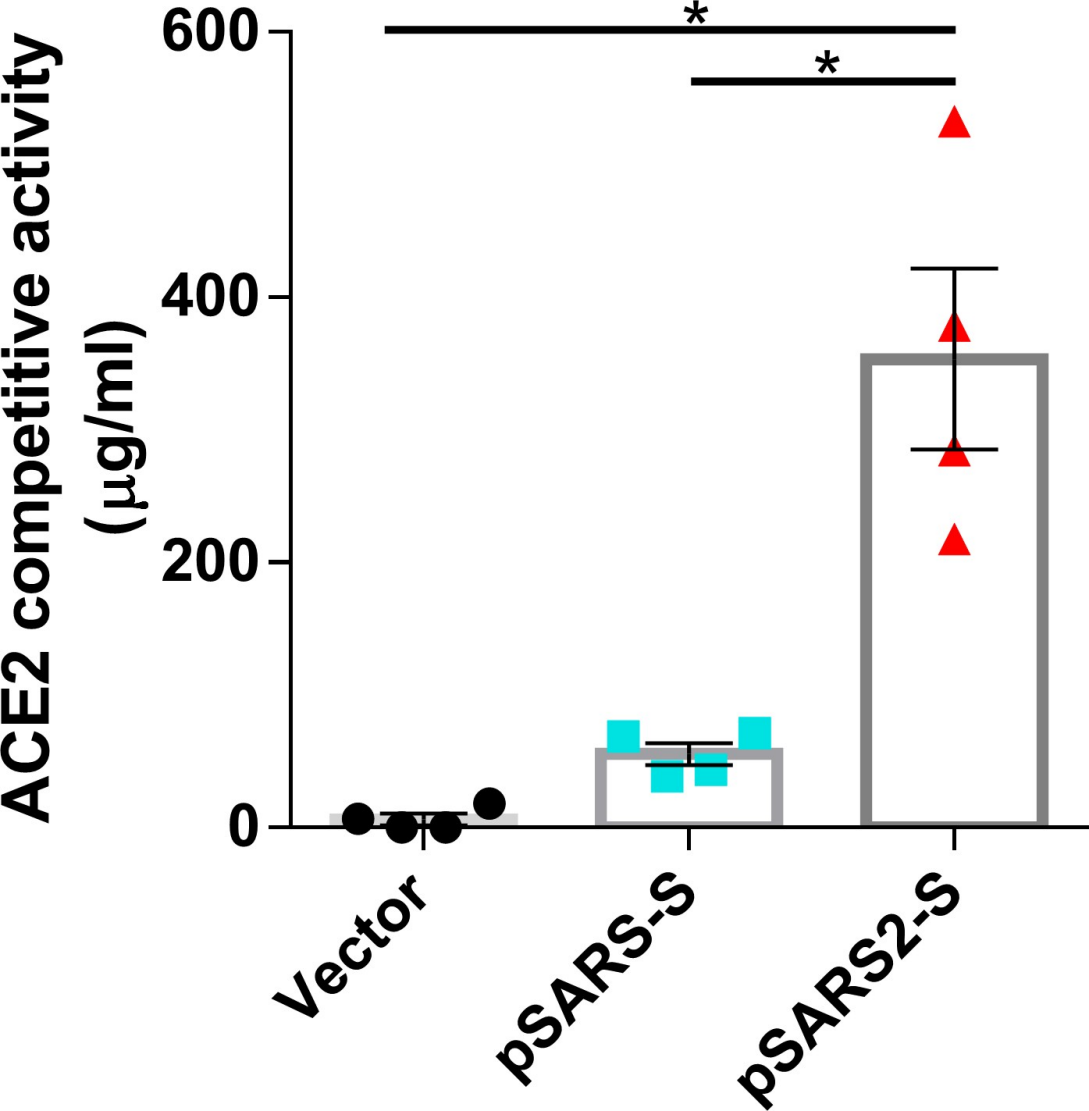
Competitive activity of immunized mouse sera against the RBD/ACE2 interaction. BALB/c mice (n = 4 per group) were intramuscularly immunized twice at a 3-week interval with 100 μg of vector, pSARS-S or pSARS2-S, followed by electroporation. Serum samples were collected at week 8 after the first immunization. Serum antibodies that compete with ACE2 for RBD binding were evaluated by competitive SARS-CoV-2 serology assay. The competitive activity of the mouse sera is expressed as the equivalent level of anti-RBD (SARS-CoV-2 spike protein) antibody (reference antibody). Antibody titers are presented as the mean ± SEM. *p<0.05 by the Mann-Whitney test.

To examine the ability of serum antibodies to interfere with the ACE2-RBD interaction, we performed a competitive SARS-CoV-2 serology assay. In this assay, serum antibodies were added to ELISA plates precoated with SARS-CoV-2 RBD protein, followed by adding of human ACE2 protein. A specific neutralizing antibody against SARS-CoV-2 RBD was used as a reference. As shown in Fig 3, serum antibodies from pSARS2-S immunized mice bound to the RBD and blocked ACE2 binding, which was equivalent to approximately 353 μg/mL reference antibody, whereas the pSARS-S sera was equivalent to 56 μg/mL reference antibody. Therefore, our data suggested that pSARS2-S immunization could induce competitive antibodies that efficiently block the binding of SARS-CoV-2 RBD to the ACE2 receptor. This result was consistent with ELISA titer against SARS-CoV2 RBD (Fig 2D), and supported by the study on the difference of RBD sequences between SARS-CoV and SARS-CoV-2 [24].

### SARS-CoV-2 S DNA vaccine induced long-term humoral immunity and offered cross-protection against the SARS-CoV-2 with D614G mutation

**Fig 4:**
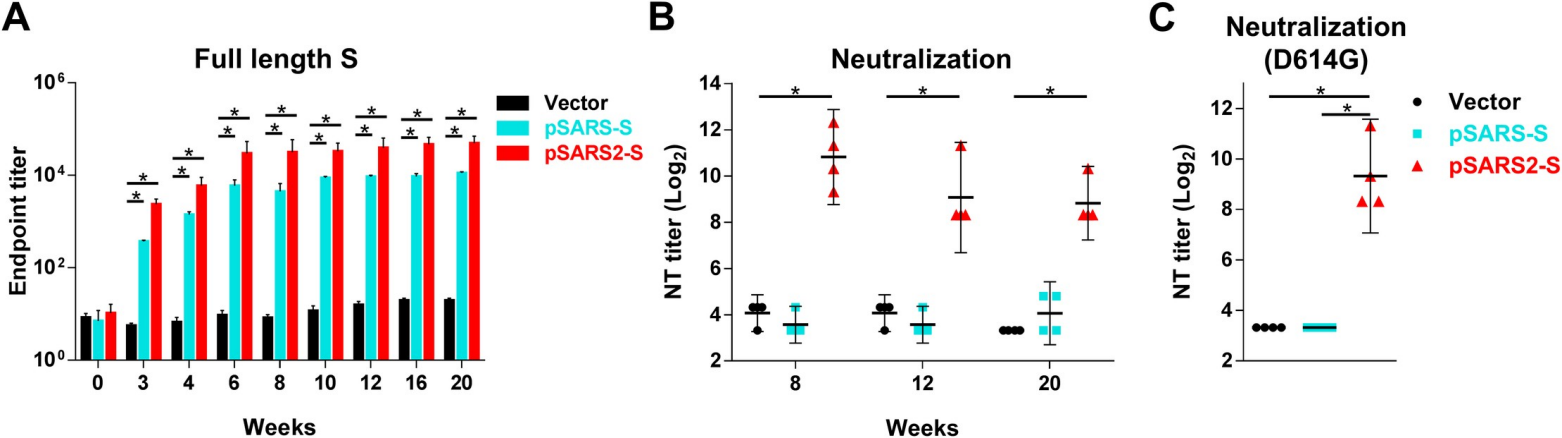
SARS-CoV-2 S DNA vaccine induced long-term humoral immunity and cross-protection against the SARS-CoV-2 with D614G mutation. BALB/c mice (n = 4 per group) were intramuscularly immunized three times at a 3-week interval with 100 μg of vector, pSARS-S or pSARS2-S, followed by electroporation. Serum samples were collected at the indicated time points after the first immunization. (A) Antibodies against the SARS-CoV-2 full-length spike protein were evaluated by ELISA. (B, C) Vaccine-induced neutralizing activity against SARS-CoV-2 with D614 or G614 genotypes was evaluated by neutralization assay. Antibody titers are presented as the mean ± SEM, and neutralization titers are expressed as the geometric mean with a 95% confidence interval. *p<0.05 by the Mann-Whitney test.

Notably, long-term maintenance of IgG antibody titers against full-length S was observed at 20 weeks after the first immunization (Fig 4A). The geometric mean titers (log_2_) for SARS-CoV-2 neutralizing antibody reached 10.8 at week 8 after pSARS2-S immunizations, slightly decreasing to 9.1 at week 12 and to 8.8 at week 20 (Fig 4B). These results suggested that pSARS2-S immunization confers a long-lasting humoral response against SARS-CoV-2. Furthermore, pSARS2-S DNA vaccine with D614 genotype induced neutralizing antibody response against the virus containing D614G mutation (Fig 4C), which is similar to neutralization titers against D614 genotype. Therefore, pSARS2-S is able to confer the cross-protection against the most prevalent and dominant D614G variant of the SARS-CoV-2.

### Induction of Th1- or Th2-biased responses

**Fig 5:**
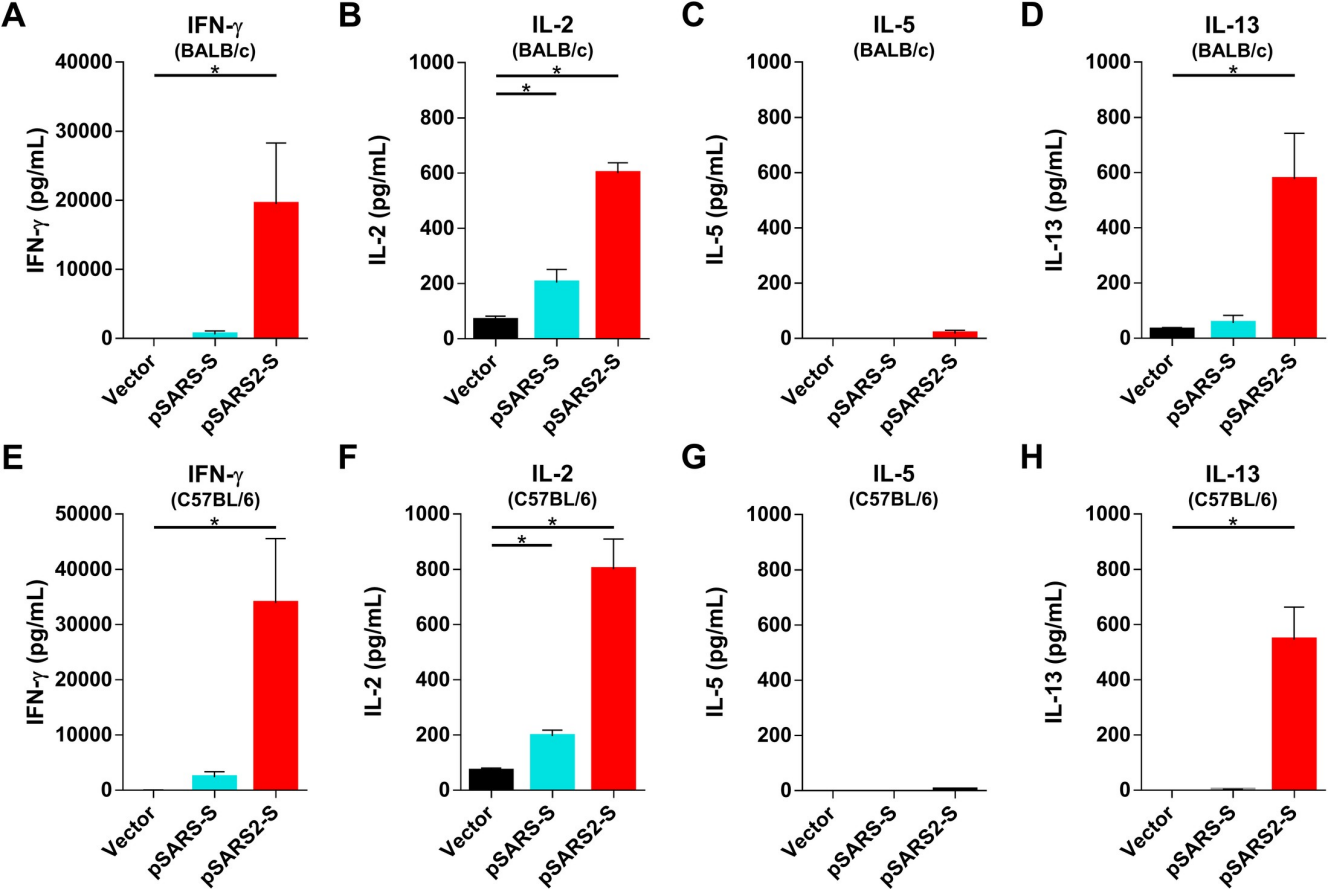
T cell response in mice after immunization with pSARS-S and SARS2-S DNA vaccines. BALB/c (A-D) and C57BL/6 (E-H) mice (n = 4 per group) were intramuscularly immunized twice at a 3-week interval with 100 μg of vector, pSARS-S or pSARS2-S, followed by electroporation. Splenocytes were collected at week 4 after the first immunization, and the levels of secreted IFN-γ (A, E), IL-2 (B, F), IL-5 (C, G) and IL-13 (D, H) were evaluated after restimulation with recombinant SARS-CoV-2 S protein. Antibody titers are presented as the mean ± SEM. *p<0.05 by the Mann-Whitney test.

Effector CD4^+^ T cells can be subdivided into two main functional subsets, Th1 and Th2, based on the secreted cytokines upon activation. Th1 cells produce inflammatory cytokines (IFN-γ) and participate in cell-mediated immune responses toward intracellular bacteria and viruses, whereas Th2 cells secrete mainly help B cells produce antibodies but also promote eosinophil-mediated immunity (IL-5 and IL-13), resulting in humoral or allergic responses [25, 26]. Moreover, Th2 cell-dependent mechanisms may contribute to vaccine-associated enhanced respiratory disease (VAERD), as shown by studies of SARS-CoV vaccine candidates [27, 28], which highlight that a balanced T cell response is critical for safe COVID-19 vaccine development [29]. To address this issue, BALB/c and C57BL/6 mice were immunized with vector, pSARS-S and pSARS2-S twice at a 3-week interval. Mice were sacrificed 7 days after the second immunization, and splenocytes were stimulated with SARS-CoV-2 S protein (5 μg/mL) for 3 days. In BALB/c mice (Fig 5A–5D), the secretion of the Th1 type cytokines IFN-γ (19641.3 pg/mL ± 8823.5) and IL-2 (599.5 pg/mL ± 37.7) was high after stimulation with S protein in the pSARS2-S immunization group, but very low levels of the Th2 type cytokines IL-5 (18.1 pg/mL ± 11.8) and IL-13 (567.2 pg/mL ± 166.2) were detected. Similar results were observed in C57BL/6 mice, pSARS2-S immunization group induced higher amount of IFN-γ (33918.8 pg/mL ± 11646.1) and IL-2 (800.3 pg/mL ± 109.5) than that of IL-5 (4.6 pg/mL ± 2.9) and IL-13 (545.7 pg/mL ± 117.4) (Fig 5E–5H). These data suggested that pSARS2-S could induce Th1-biased immune responses.

### Prophylactic efficacy of DNA vaccines against SARS-CoV-2 challenge

**Fig 6:**
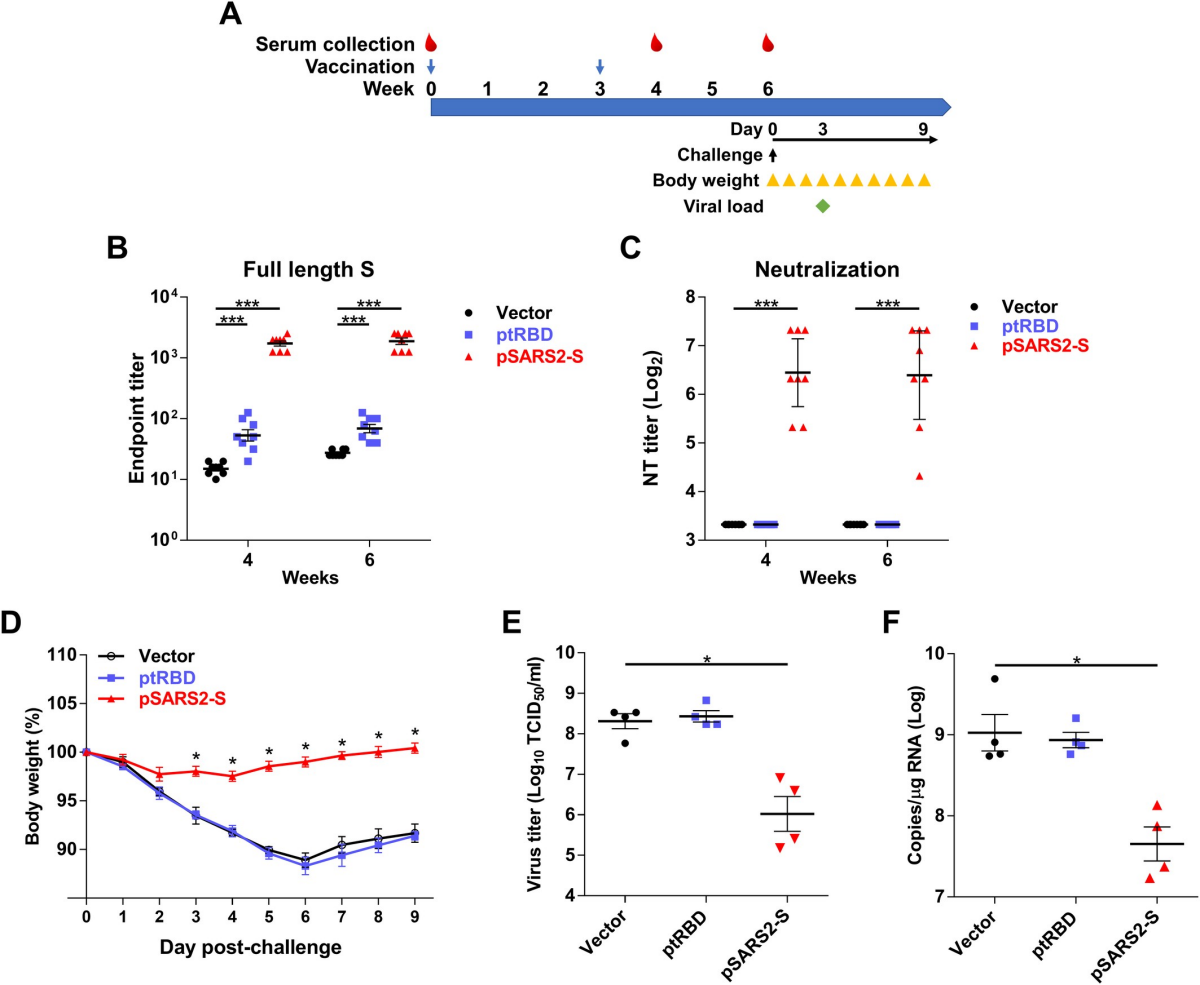
Prophylactic efficacy of SARS-CoV-2 S DNA vaccine in SARS-CoV-2-infected hamsters. (A) Time course of DNA vaccination and SARS-CoV-2 challenge. Syrian hamsters were intramuscularly immunized twice at a 3-week interval with 100 μg of control, pSARS-S or pSARS2-S, followed by electroporation. Serum samples were collected by retroorbital blood sampling at weeks 4 and 6 after the first immunization. At 4 weeks after the second immunization, Syrian hamsters were intranasally challenged with 10^5^ TCID_50_ SARS-CoV-2. (B) Antibodies against the SARS-CoV-2 full-length spike protein were evaluated by ELISA. (C) Vaccine-induced neutralizing activity against SARS-CoV-2 was evaluated by neutralization assay. (D) Body weight change (%) of the hamsters was recorded every day after SARS-CoV-2 challenge. Virus titers (E) and viral RNA copies (F) in the lungs of SARS-CoV-2-infected hamsters at 3 days postchallenge were determined by TCID_50_ assay and qRT-PCR, respectively. Antibody titers are presented as the mean ± SEM, and neutralization titers are expressed as the geometric mean with a 95% confidence interval. *p<0.05, ***p<0.001 by the Mann-Whitney test.

To explore the protective efficacy of pSARS2-S vaccination, Syrian hamsters were immunized twice with 100 μg DNA at a 3-week interval and intranasally challenged with SARS-CoV-2 virus at week 7 (Fig 6A). After immunization, week 4 and week 6 sera were collected for anti-Spike antibody (IgG) titer and neutralizing antibody titer analyses. Immunization with pSARS2-S elicited higher levels of anti-Spike antibody titers than ptRBD immunization at week 4 (1584.9 vs. 50.1) and week 6 (1995.3 vs. 63.1) in hamsters (Fig 6B). Consistent with the anti-Spike antibody titers, the sera of pSARS2-S-immunized hamsters raised very high neutralizing antibody titers (6.5 at week 4 and 6.4 at week 6 (log_2_)), but the sera of vector- or ptRBD-immunized hamsters did not (Fig 6C). After challenge with SARS-CoV-2, hamster body weight was monitored every day. Previous studies revealed that the viral titer in hamster lung reached a high level at 3 days postchallenge [30]. Therefore, half of the hamsters from each group were sacrificed on day 3 and the viral load in the lung was analyzed. The body weight of vector-vaccinated hamsters decreased gradually, and the percentage of body weight lost was 11.1% at 6 days postchallenge. In contrast, pSARS2-S immunization protected hamsters from body weight loss (Fig 6D). Furthermore, the infectious virus titers and number of viral RNA copies in the pSARS2-S group showed 2.29 and 1.37 log_10_ reductions compared with the vector control group (Fig 6E and 6F). These results suggested that pSARS2-S immunization confers protection against SARS-CoV-2 infection in Syrian hamsters.

## Discussion

More than 80 COVID-19 vaccine clinical trials have been launched, and immunogenicity and viral challenge studies in animals are critical steps in the vaccine development processes. DNA vaccines for SARS-CoV-2 infection have been intensively developed for different delivery approaches. Electroporation is a promising approach that can enhance DNA delivery and the antigenicity of immunogens *in vivo*. Two DNA vaccine studies have been reported, by Yu et al. [13] and Smith et al. [12]. Yu et al. found that rhesus macaques immunized with naked DNA encoding full-length S protein (without electroporation) exhibited >3.1 log_10_ reductions in viral loads in bronchoalveolar lavage after challenge compared with controls. Smith et al. found that immunization of mice and guinea pigs with INO-4800 (encoding full-length S protein) with electroporation could elicit neutralizing antibodies against SARS-CoV-2 infection and block S protein binding to the ACE2 receptor but did not provide animal challenge data. In this report, we evaluated different variants for DNA vaccine candidates and found that the full-length S protein (pSARS2-S) is the most suitable for further immunological study. Although SARS-CoV and SARS-CoV-2 share 76% homology in their S proteins [31, 32], pSARS-S immunization cannot induce antibodies against the RBD of the SARS-CoV-2 S protein or neutralizing antibody titers against SARS-CoV-2 infection (Fig 2). Indeed, anti-RBD antibodies play important roles in blocking viral infection. However, immunization with RBD alone (ptRBD) generated high neutralizing antibody titers in mice but not in hamsters (Figs 2H and 6C). We speculated that this result may be due to the signal peptide (tissue-plasminogen activator) failing to facilitate the secretion of the RBD protein in hamsters. The detailed mechanism requires further study in the future. We also noted that ptRBDTM (encoding a fragment from the RBD to the transmembrane domain of S) immunization induced lower neutralizing antibody titers than ptRBD immunization (Figs 2H and S1C). The results may reflect the unstable structure of the RBD-TM protein. To further investigate the Th1/Th2 immune responses, splenocytes from immunized mice were stimulated with SARS-CoV-2 S protein. We found that pSARS2-S immunization induced strong Th1-biased immune responses with higher levels of IFN-γ-secretion after stimulation (Fig 5), but pSARS-S immunization induced only low levels of IFN-γ-secretion. These data indicated that the cross-reactivity of T cell responses between the spike protein of SARS-CoV and SARS-CoV-2 is not high (Fig 5). Furthermore, IFN-γ ELISPOT assay revealed that less T cells responses were detected against S1 region in C57BL/6 and BALB/c mice immunized with pSARS-S (S2 Fig), which is also supported by Smith’s study [12]. These data indicated that the SARS vaccine may not provide full protective effect against SARS-CoV-2 infection. The development of DNA vaccines is important for a rapid response to the pandemic coronavirus infection. Hence, the success of DNA vaccine against SARS-CoV-2 could be applied to other emergent infectious diseases.

The different constructs used in the SARS-SoV-2 DNA vaccines induced different levels of neutralizing antibody titers. The tPA leader sequence has been used to increase antigen expression and secretion in DNA vaccines [21, 33–35]. However, the tPA leader sequence did not significantly increase the antibody titers in this report. To further increase vaccine efficacy, different leader sequences could be used to replace the native leader sequence of the spike protein. The IgE leader sequence was used in the INO-4800 DNA vaccine and MERS-CoV vaccine [5, 12]. In addition, the modified spike protein sequence may also increase the immunogenicity of the vaccine. A stabilized spike protein has been designed by mutating the furin site and other regions to generate a prefusion structure (S-2P) that can increase the expression of the spike protein ~10-fold [36]. An ideal DNA vaccine should optimize plasmid DNA construct and delivery system to increase protein expression level.

Animal models are critical for COVID-19 vaccine development. Several animal models have been used to evaluate the efficacy of COVID-19 vaccines, including nonhuman primates [13, 37], human ACE2 transgenic mice [38] and Syrian hamsters [39]. Syrian hamster ACE2 has high similarity to human ACE2, and its binding affinity to the S protein of SARS-CoV-2 were predicted higher than mouse ACE2 [40]. This is the reason why human ACE2 transgenic mice were used as SARS-CoV-2 challenge model, but not wildtype mice. SARS-CoV-2 transmission studies have shown that the virus can efficiently infect naive hamsters through direct contact or via aerosols [41]. Intranasal infection of SARS-CoV-2 can replicate and induce pathogenesis in the lungs of Syrian hamsters [30]. This study and previous reports also showed SARS-CoV-2 infection caused approximately 10% reduction from initial body weight of hamsters. Clinical manifestations of patients with COVID-19, including changes in smell and taste, and severe respiratory distress, might be accompanied with weight loss (>5% reduction from baseline), which were associated with longer disease duration [42]. These findings indicate that the Syrian hamster is also a suitable animal model for the evaluation of COVID-19 vaccines. Based on the availability of animal model, we chose hamsters as SARS-CoV-2 challenge model to evaluate vaccine efficacy. Our data showed that immunization with pSARS2-S, but not ptRBD, can induce high titers of anti- Spike IgG antibody and neutralizing antibody titers (Fig 6B and 6C). Accordingly, pSARS2-S-immunized hamsters, but not ptRBD-immunized hamsters, generated immune responses against SARS-CoV-2 challenge. We noted that ptRBD immunization could induce high levels of neutralizing antibody titers in mice but not in hamsters. The conflicting results may be because RBD alone is not stably expressed in hamsters. The hamster model was also used to evaluate the adenovirus serotype 26 (Ad26) vector-based COVID-19 vaccine. A single immunization with the Ad26 vector-based vaccine expressing a stabilized SARS-CoV-2 spike protein that elicited neutralizing antibody responses and protected against SARS-CoV-2 infection induced weight loss, partial mortality and viral replication in the lung [39]. These results indicated that the hamster model is suitable for evaluating the efficacy of COVID-19 vaccines.

Plenty of SARS-CoV-2 vaccine development efforts is based on the research experience of MERS-CoV and SARS-CoV. Globally, several COVID-19 vaccines have been approved for emergent used in December 2020, including RNA- and adenovirus vector-based COVID-19 vaccines. The advantages and disadvantages of different vaccine platforms have been discussed [43]. Adenovirus vector vaccines may elicit stronger immune response than DNA and mRNA vaccines, but their vaccine efficacy could reduce through pre-existing immunity against Ad vectors [44]. Compared to DNA vaccine, mRNA vaccine needs ultralow temperature for storage and transportation [45]. Hence, DNA vaccine could be a potential vaccine platform especially during emergency use.

Our study suggests the possibility of a DNA vaccine for human use. Further studies could investigate the efficacy of this DNA vaccine delivered using intradermal (ID) injection, which is more convenient for clinical application, because the needle for IM injection is approximately 18 mm deep in humans and affects more tissues than ID injection by EP. Moreover, vaccine efficacy should be tested in aged mice as a model for elderly humans, because this population is particularly severely affected when infected by SARS-CoV-2. Previous studies showed that Th2 cell response have been associated with enhanced respiratory disease (VAERD) following the vaccination of inactivated virus vaccines against RSV [46], measles virus [47] and SARS-CoV [27, 48]. In contrast, there are less severe cases of SARS-CoV resulting from induction of Th1 cell response being reported [49]. Therefore, the strong Th1-biased immune responses induced by the DNA vaccine suggest that side effects are unlikely to be a major issue [50]. Besides this, concerns about protein-based vaccines have been raised due to the use of aluminum salt or oil-in-water emulsion-type adjuvants, which lead to Th2-biased immune responses and increase potential side effects [51, 52]. In summary, the COVID-19 DNA vaccine may play a major role in controlling pandemic COVID-19 in near future.

## Supporting information

S1 Fig Antibody response in mice after immunization with SARS-CoV-2 spike variants at week 4. BALB/c mice (n = 4 per group) were intramuscularly immunized twice at a 3-week interval with 100 μg of vector, ptRBD, ptRBDTM, ptSARS2-S or ptSdTM, followed by electroporation. Serum samples were collected at weeks 4 after the first immunization. (A, B) Antibodies against the SARS-CoV-2 full-length spike protein and RBD were assessed by ELISA. (C) Vaccine-induced neutralizing activity against SARS-CoV-2 was evaluated by neutralization assay. Antibody titers are presented as the mean ± SEM, and neutralization titers are expressed as the geometric mean with a 95% confidence interval. *p<0.05 by the Mann-Whitney test.
(TIF)

S2 Fig T cell response in mice after immunization with pSARS-S and SARS2-S DNA vaccines. C57BL/6 (A) and BALB/c (B) mice (n = 4 per group) were intramuscularly immunized twice at a 3-week interval with 100 μg of vector, pSARS-S or pSARS2-S, followed by electroporation. Splenocytes were collected at week 5 after the first immunization, and T cell responses were analyzed by IFN-γ ELISpot assay following stimulation of indicated peptides and recombinant proteins for 48 h.
(TIF)
