# Correction: DNA vaccination induced protective immunity against SARS CoV-2 infection in hamsterss

**DOI:** 10.1371/journal.pntd.0011045

**Published:** 2023-01-06

**Authors:** Kit Man Chai, Tsai-Teng Tzeng, Kuan-Yin Shen, Hung-Chun Liao, Jhe-Jhih Lin, Mei-Yu Chen, Guann-Yi Yu, Horng-Yunn Dou, Ching-Len Liao, Hsin-Wei Chen, Shih-Jen Liu

The word "hamsters" is misspelled in the article title. The correct title is: DNA vaccination induced protective immunity against SARS CoV-2 infection in hamsters. The correct citation is: Chai KM, Tzeng T-T, Shen K-Y, Liao H-C, Lin J-J, Chen M-Y, et al. (2021) DNA vaccination induced protective immunity against SARS CoV-2 infection in hamsters. PLoS Negl Trop Dis 15(5): e0009374. https://doi.org/10.1371/journal.pntd.0009374
