# Supplementary figures and images for: DNA vaccination induced protective immunity against SARS CoV-2 infection in hamsterss
Source: PLoS Negl Trop Dis. 2021 May 27;15(5):e0009374. doi: 10.1371/journal.pntd.0009374 (PMC8158926; doi:10.1371/journal.pntd.0009374)

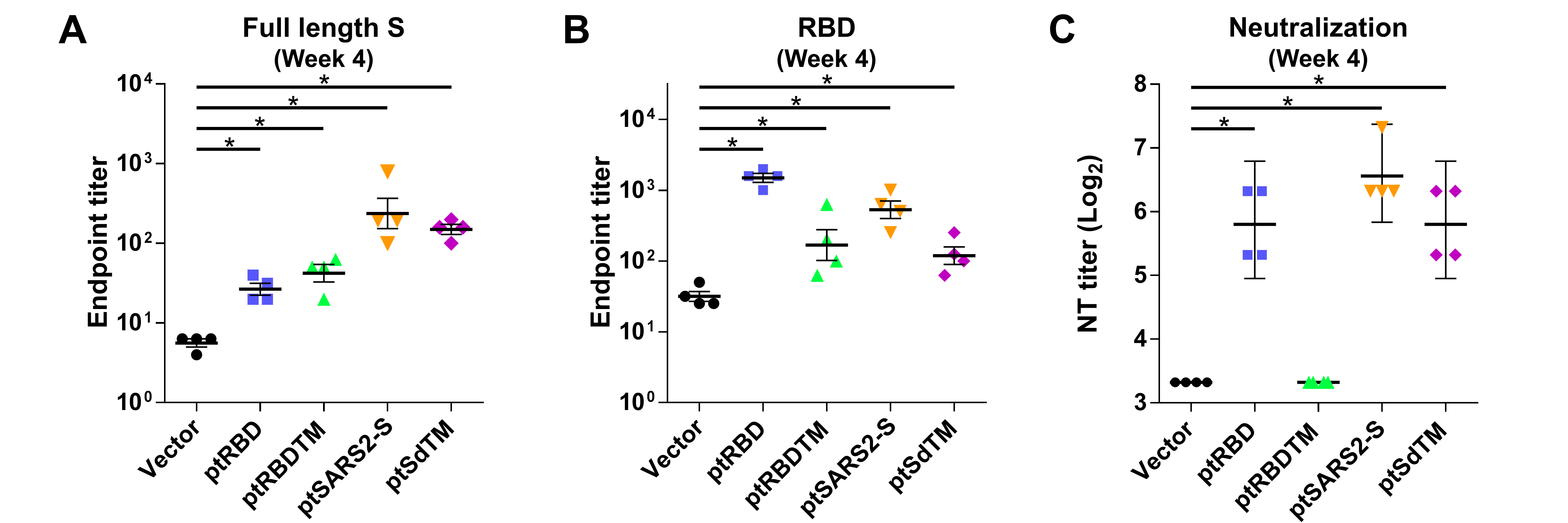

Supplement: S1 Fig — BALB/c mice (n = 4 per group) were intramuscularly immunized twice at a 3-week interval with 100 μg of vector, ptRBD, ptRBDTM, ptSARS2-S or ptSdTM, followed by electroporation. Serum samples were collected at weeks 4 after the first immunization. (A, B) Antibodies against the SARS-CoV-2 full-length spike protein and RBD were assessed by ELISA. (C) Vaccine-induced neutralizing activity against SARS-CoV-2 was evaluated by neutralization assay. Antibody titers are presented as the mean ± SEM, and neutralization titers are expressed as the geometric mean with a 95% confidence interval. *p<0.05 by the Mann-Whitney test. (TIF) [file pntd.0009374.s001.tif]

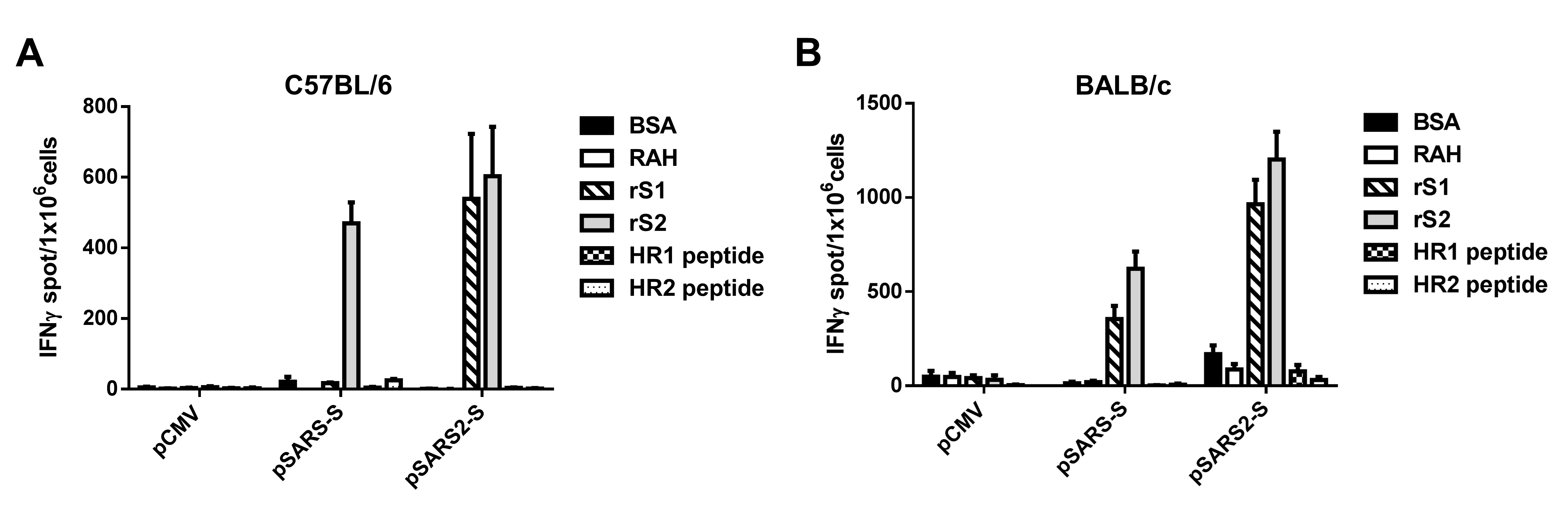

Supplement: S2 Fig — C57BL/6 (A) and BALB/c (B) mice (n = 4 per group) were intramuscularly immunized twice at a 3-week interval with 100 μg of vector, pSARS-S or pSARS2-S, followed by electroporation. Splenocytes were collected at week 5 after the first immunization, and T cell responses were analyzed by IFN-γ ELISpot assay following stimulation of indicated peptides and recombinant proteins for 48 h. (TIF) [file pntd.0009374.s002.tif]
